# Comparison of machine‐learning models for the prediction of 1‐year adverse outcomes of patients undergoing primary percutaneous coronary intervention for acute ST‐elevation myocardial infarction

**DOI:** 10.1002/clc.24157

**Published:** 2023-09-18

**Authors:** Saeed Tofighi, Hamidreza Poorhosseini, Yaser Jenab, Mohammad Alidoosti, Mohammad Sadeghian, Mehdi Mehrani, Zhale Tabrizi, Parisa Hashemi

**Affiliations:** ^1^ Tehran Heart Center, Cardiovascular Diseases Research Institute Tehran University of Medical Sciences Tehran Iran; ^2^ Department of Radiology Iran University of Medical Sciences Tehran Iran; ^3^ School of Medicine Shahid Beheshti University of Medical Sciences Tehran Iran

**Keywords:** machine learning, myocardial infarction, percutaneous coronary intervention

## Abstract

**Background:**

Acute ST‐elevation myocardial infarction (STEMI) is a leading cause of mortality and morbidity worldwide, and primary percutaneous coronary intervention (PCI) is the preferred treatment option.

**Hypothesis:**

Machine learning (ML) models have the potential to predict adverse clinical outcomes in STEMI patients treated with primary PCI. However, the comparative performance of different ML models for this purpose is unclear.

**Methods:**

This study used a retrospective registry‐based design to recruit consecutive hospitalized patients diagnosed with acute STEMI and treated with primary PCI from 2011 to 2019, at Tehran Heart Center, Tehran, Iran. Four ML models, namely Gradient Boosting Machine (GBM), Distributed Random Forest (DRF), Logistic Regression (LR), and Deep Learning (DL), were used to predict major adverse cardiovascular events (MACE) during 1‐year follow‐up.

**Results:**

A total of 4514 patients (3498 men and 1016 women) were enrolled, with MACE occurring in 610 (13.5%) subjects during follow‐up. The mean age of the population was 62.1 years, and the MACE group was significantly older than the non‐MACE group (66.2 vs. 61.5 years, *p* < .001). The learning process utilized 70% (*n* = 3160) of the total population, and the remaining 30% (*n* = 1354) served as the testing data set. DRF and GBM models demonstrated the best performance in predicting MACE, with an area under the curve of 0.92 and 0.91, respectively.

**Conclusion:**

ML‐based models, such as DRF and GBM, can effectively identify high‐risk STEMI patients for adverse events during follow‐up. These models can be useful for personalized treatment strategies, ultimately improving clinical outcomes and reducing the burden of disease.

## Graphical abstract

This study assessed the performance of four machine learning models for predicting adverse outcomes in acute ST‐elevation myocardial infarction (STEMI) patients undergoing primary percutaneous coronary intervention. Among the models tested, the Distributed Random Forest and Gradient Boosting Machine exhibited superior predictive accuracy for major adverse cardiovascular events during a 1‐year follow‐up. These models hold the potential for enhancing personalized treatment strategies and improving clinical outcomes in high‐risk STEMI patients.

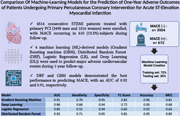

## 1 BACKGROUND

Acute ST‐elevation myocardial infarction (STEMI) is a major cause of morbidity and mortality worldwide, with primary percutaneous coronary intervention (PCI) being the preferred treatment option.^1^ Despite the significant advances in treatment, some patients still experience adverse clinical outcomes, such as cardiogenic shock, heart failure, and death. Early identification of patients at high risk of adverse clinical outcomes is critical for improving clinical outcomes and reducing the burden of disease. Machine learning (ML) models have shown great potential in identifying high‐risk patients and predicting adverse clinical outcomes in various medical conditions, including STEMI.^2^ However, the comparative performance of various ML models for predicting adverse clinical outcomes of STEMI patients treated with primary PCI remains unclear. Therefore, in this study, we aim to compare the performance of various ML models, including Logistic Regression (LR), Distributed Random Forest (DRF), Gradient Boosting Machines (GBMs), and Deep Learning (DL) for predicting adverse clinical outcomes of STEMI patients treated with primary PCI. By leveraging large data sets of clinical and imaging data, we aim to identify the most accurate and reliable machine‐learning model for predicting adverse clinical outcomes of STEMI patients. Our findings may help improve the early identification of high‐risk patients and facilitate personalized treatment strategies to improve clinical outcomes and reduce the burden of disease.

## 2 MATERIALS AND METHODS

### 2.1 Study design and patient selection

In this retrospective study conducted in Tehran Heart Center (THC), Tehran, Iran, from 2011 to 2019, all consecutive hospitalized patients diagnosed with acute STEMI, who were treated by primary PCI, were recruited. A total number of 6340 patients entered at first with a diagnosis of STMEI, 1286 subjects were excluded from the study as they were not undergone PCI and were managed either with medical treatment or scheduled for coronary artery bypass grafting (CABG) surgery regarding their coronary disease complexity. Also, 540 patients were excluded due to remarkable missing data in our registration system. Eventually, 4514 subjects were enrolled in the study for further analysis. All patients had at least 1‐year postdischarge follow‐up. In general, the follow‐ups were conducted three times: 1‐, 6‐, and 12‐month following hospital discharge. Nonetheless, our analysis exclusively utilized data from 1‐year follow‐up assessments as the final outcome. All employed models were trained with the specific objective of predicting major cardiovascular events occurring within this 1‐year timeframe.

### 2.2 Study endpoints

The main endpoint of the present study was a composite of the major adverse cardiovascular events (MACEs) during 1‐year follow‐up, including myocardial infarction, emergent revascularization, hemodynamic instability, and all‐cause mortality. Hemodynamic instability was defined as low systolic blood pressure that needs inotrope therapy and/or mechanical ventilation in the course of their admission.

### 2.3 Statistical analysis

As it is illustrated in Table 1, continuous and categorical variables were represented as mean and frequencies, respectively. Statistical analysis was performed with independent samples *t* test for continuous numerical variables. Also, the *χ* ^2^ test and Fisher exact test were done to evaluate the relationship between categorical variables and final adverse outcomes as appropriate. The significance level for all of the statistical analyses was determined as a *p* value of lower than .05.

**Table 1:** Demographic, diagnostic, and procedural characteristics of the study patients.

	Total population (95% CI for mean)	Non‐MACE group (*n* = 3904)	MACE group (*n* = 610)	*p* Value
Age (year)	62.1 (61.8–62.5)	61.5	66.2	<.001
Sex (male, %)	77.5	78.0	74.3	.042
BMI (kg/m^2^)	27.2 (27.8–28.5)	27.9	26.6	.032
Risk factors				
FH (%)	17.5	18.2	13.4	.004
DM (%)	41.8	40.2	51.8	<.001
HTN (%)	46.3	45.7	50.5	.029
DLP (%)	53.7	49.7	52.2	.032
C/S (%)	46.0	45.2	46.1	.727
O/A (%)	16.2	16.1	16.6	.813
Past medical history				
Previous STEMI (%)	4.1	3.7	7.0	<.001
Previous non‐STEMI (%)	2.0	1.8	3.6	.005
CHF (%)	2.9	2.1	8.2	<.001
DHF hospitalization (%)	1.5	5.2	0.9	<.001
CVA (%)	3.5	2.8	7.4	.001
COPD (%)	1.9	1.8	2.6	.150
CKD (%)	1.8	1.2	6.1	<.001
ESRD (on dialysis) (%)	0.2	0.1	1.0	.001
Prior CPR (%)	2.0	1.7	3.9	.001
Previous PCI (%)	10.7	10.2	14.1	.006
Previous CABG (%)	5.6	5.3	7.5	.029
Echocardiography				
LVEF (%)	41.2 (40.9–41.4)	41.7	38.0	<.001
Significant VHD (%)	1.3	0.9	4.1	<.001
RV function (%)				
Normal	66.8	68.2	57.5	<.001
Mildly impaired	27.0	26.6	29.8	
Moderately impaired	5.7	4.9	11.1	
Severely impaired	0.5	0.3	1.5	
Electrocardiogram				
AF rhythm (%)	1.0	0.7	2.6	<.001
High degree AVB (%)	0.9	0.7	1.8	.017
LBBB (%)	1.2	1.1	2.1	.046
RBBB (%)	3	2.9	3.9	.163
LVH criteria (%)	0.1	0.1	0.2	.582
Q wave (%)	29.4	28.9	32.3	.094
Frequent PVC (%)	1.0	0.9	1.3	.373
Laboratory tests				
WBC (×10^3^/dL)	11.34 (11.2–11.4)	11.1	12.3	<.001
Hb (g/dL)	14.9 (14.8–15.0)	15.0	14.4	<.001
PLT (×10^6^/dL)	243.3 (241.1–245.5)	242.9	246.2	.372
Cr (mg/dL)	1.01 (1.00–1.03)	0.9	1.1	<.001
Procedural parameters				
P‐D time (min)	530.2 (507.7–552.7)	534.4	503.3	.355
D‐D time (min)	77.8 (75.8–79.8)	78.3	74.7	.229
P‐D time (min)	608.0 (585.3–630.8)	612.7	578.0	.307
Lesion length (mm)	28.0 (27.7–28.9)	27.6	30.8	.003
IIb/IIIa in administration (%)	50.8	51.4	47.0	.045
In‐stent thrombosis (%)	1.0	1.0	1.1	1.000

Abbreviations: AF, atrial fibrillation; AVB, atrioventricular block; BMI, body mass index; C/S, cigarette smoker; CABG, coronary artery bypass grafting; CHF, congestive heart failure; COPD, chronic obstructive pulmonary disease; Cr, creatinine; CVA, cerebrovascular events; D‐D time, door‐to‐device time; DHF, decompensated heart failure; DLP, dyslipidemia; DM, diabetes mellitus; ESRD, end‐stage renal disease; FH, family history; Hb, hemoglobin; HTN, hypertension; LBBB, left bundle branch block; LVEF, left ventricular ejection fraction; LVH, left ventricular hypertrophy; O/A, opium addiction; P‐D time, pain‐to‐device time; P‐D time, pain‐to‐door time; PLT, platelets; PVC, premature ventricular contraction; RBBB, right bundle branch block; RV, right ventricle; VHD, valvular heart disease; WBC, white blood cells.

### 2.4 Data extraction and processing

Demographics and clinical and paraclinical variables were extracted from Electronic Health Records. A total number of 156 initial variables were identified for each patient. Based on our primary analysis (explained in Section 2.6), eventually, we retained 50 of the most important variables for further model developments.

### 2.5 Missing data

We had missing data in several variables for several situations. Our approach to missing data was a combination of two methods: (1) imputation or (2) data removal. If the variable was significant for the prediction process and the missing values were minimal, we used the *K*‐nearest neighbor (KNN) approach to impute data. This is a common strategy for dealing with missing data that effectively imputes the predicted values instead of the missing ones while having less of an influence on the final analysis than other traditional methods. On the other hand, if the number of missing values was substantial and the variable was not significant enough, the variable may be deleted.

### 2.6 Feature selection

The next step was to choose the best variables for model development (after data preprocessing and missing value management). In this step, which is called “feature selection,” we used two methods: first, regarding traditional statistical analysis, we determined the variables that had significant differences between the two groups; second, we utilized the more precise method, L1 regularization (or Lasso regression) method, which is one of the best methods for feature selection in data science. L1 regularization lets us find the most important variables for the prediction of final results. Using this method and the traditional analysis, we eventually determined 50 variables that were more important for model development.

### 2.7 Model development

To create prediction models for a data set of 4514 patients, the data were randomly divided into three categories: a training set comprising 56% of the total population (*n* = 2528), a validation set comprising 14% of the total population (*n* = 632), and a testing set comprising 30% of the total population (*n* = 1354). Four prediction models were developed using the R programming language, including the Gradient Boosting and DRF models, which fall under the Ensemble Machine Learning methods, a DL model, and an LR model. These models were trained using the training set, and their hyperparameters were tuned using the validation set. Finally, the models were fitted onto the testing set to determine their performance metrics, which were compared to identify the most accurate model.

### 2.8 The ensemble machine learning methods

Ensemble ML methods are a type of algorithm that employ multiple learning techniques to achieve superior predictive accuracy than that which can be achieved by using any individual learning algorithm alone. Several popular contemporary ML algorithms are, in fact, ensemble learners, including the Random Forest (RF) and GBM. Bagging, as used in RF, and boosting, as utilized by GBM, are two methods of ensembling that operate by consolidating a group of weak learners, such as a decision tree, into a single, powerful learner.

GBM is a popular machine‐learning algorithm used for both regression and classification problems.^3^ It is an ensemble method that combines the predictions of multiple weaker models to make a more accurate prediction. The basic idea behind gradient boosting is to train a sequence of models, where each subsequent model learns to correct the mistakes made by the previous model. In other words, each new model is trained to minimize the errors of the previous model. The final prediction is then made by combining the predictions of all the models.

DRF model is another algorithm belonging to the ensemble ML method that uses an ensemble of decision trees to perform classification or regression tasks.^4^ It is called “distributed” because the model is trained using a distributed computing system, which allows for parallel processing of the data. RFs are known for their high accuracy and robustness against overfitting, as well as their ability to handle large data sets. They work by building multiple decision trees on randomly sampled subsets of the data and combining their predictions to make a final prediction. In a DRF, these decision trees are built on different nodes of the computing system, allowing for faster and more efficient training.

### 2.9 The LR model

The LR model is a typical method for predicting the class of a categorical type variable (the “target variable”) using independent variables (the “predictors”). LR employs the log odds (the logarithm of the odds) to estimate the probability of one target out of two possible outcomes (binary LR), and the class of the target variable is determined based on this probability.^5^

### 2.10 The DL model

The DL model utilizes weights assigned during a learning process to connect nodes in consecutive layers. The output from the layers is the probability of the target variable, which is then converted to the predicted class based on previous learnings. Although the DL method is primarily used for developing prediction models on large data sets, it can also be applied to data sets of any size. In such cases, techniques should be implemented to augment the training data set and improve the final estimations.^5^ The DL model employed in this study is based on a multilayer perception (MLP) method, which is particularly suited to tabular data. The model comprises four layers, including an input layer, two hidden layers (each containing ten nodes), and an output layer.

### 2.11 Model evaluation

We evaluated the predictive power of the models on a single testing data set. Many factors should be considered for this purpose (called “performance metrics”), such as the area under the curve (AUC) of the receiver operating characteristic (ROC) curve, accuracy, sensitivity, specificity, F1‐score, and the Matthews Correlation Coefficient (MCC) of the model. The “f1‐Score” is a statistical index that represents the harmonic mean of the Precision and Recall measures. It is widely used in evaluating the performance of classification models. On the other hand, The MCC is a more powerful index for assessing the accuracy of a system, ranging between −1 and +1, where +1 indicates the most accurate prediction without any chance effect or errors. The formula for MCC is based on Formula‐1, and it provides a comprehensive measure of the classification system performance that considers true and false positives and negatives.

Formula 1. The MCC Equation

MCC=(TP×TN+FP×FN)/√((TP+FP)×(TP+FN)×(TN+FP)×(TN+FN)).

## 3 RESULTS

The demographic, diagnostic, and procedural characteristics of the enrolled patients are depicted in Table 1. The whole data set is divided into two groups: the MACE group (*n* = 610) versus the non‐MACE group (*n* = 3904). The mean age of the total population was 62.1 years, while people in the MACE group were significantly older than the non‐MACE group. (66.2 vs. 61.5 years, *p* < .001). Most of the population in both groups was male, with slightly higher rates in the non‐MACE group (78.0% vs. 74.3%, *p* = .042). Moreover, the mean body mass index in patients with a MACE was remarkably lower than those without any MACE in their follow‐up (26.6 vs. 27.9 kg/m^2^, *p* = .032). Regarding the prevalence of cardiovascular risk factors in group subjects, including diabetes, hypertension, dyslipidemia, and family history of premature ischemic heart diseases, all were significantly higher in the MACE group. Furthermore, people in the MACE group had more past history of different cardiovascular diseases, such as STEMI, NSTMEI, congestive heart failure (CHF), hospitalization for decompensated heart failure (DHF), cerebrovascular events (CVA), chronic kidney disease (CKD), and end‐stage renal disease (ESRD), and previous history of coronary revascularization, including PCI and CABG. In terms of cardiac function and structure, mean left ventricular ejection fraction (LVEF) was remarkably lower in the MACE group (38.0% vs. 41.7%, *p* < .001), and right ventricular function was more impaired in this group as well (*p* < .001).

The important point is that, as the primary PCI protocol for STEMI patients in our center is identical for all scenarios, all the pain‐to‐door, door‐to‐device, and pain‐to‐device times were similar between the two groups. However, the mean lesion length was higher in the MACE group compared with the non‐MACE group (27.6 vs. 30.8 mm, *p* = .003).

### 3.1 Models performance

To assess the performance of models in predicting adverse outcomes in a particular population, an evaluation of performance metrics is necessary. These metrics for our four models are presented in Table 2 and provide a basis for comparing the performance of various models. Such an analysis enables the selection of the most suitable model for predicting adverse outcomes in the given population. Obviously, the two models that were based on the ensemble methods, the GBM and the DRF models, are better than the two other models according to all parameters.

**Table 2:** Performance metrics of different machine learning models.

Model	AUC	Sensitivity	Specificity	F1 score	Accuracy	MCC
Gradient Boosting Machine	0.91	0.70	0.95	0.82	0.96	0.81
Deep Learning	0.86	0.66	0.94	0.72	0.93	0.68
Logistic Regression	0.85	0.52	0.94	0.65	0.92	0.64
Distributed Random Forest	0.92	0.71	0.95	0.81	0.95	0.80

Abbreviations: AUC, area under the curve of ROC plot; MCC, Matthew's correlation coefficient.

### 3.2 The learning curves

The learning curve is a graphical representation of the performance of ML models during training and validation of the data, and it is a crucial tool for understanding how well a model is learning from the data. The learning curve shows the relationship between the training set size and the model's performance. As the size of the training set increases, the performance of the model on the training data usually improves, while the performance on the validation set tends to plateau. This indicates that the model has learned the patterns in the training data too well, leading to overfitting, which reduces the generalization performance on the unseen validation data. Therefore, finding the right balance between the size of the training set and the complexity of the model is crucial for achieving good generalization performance.

**Figure 1:**
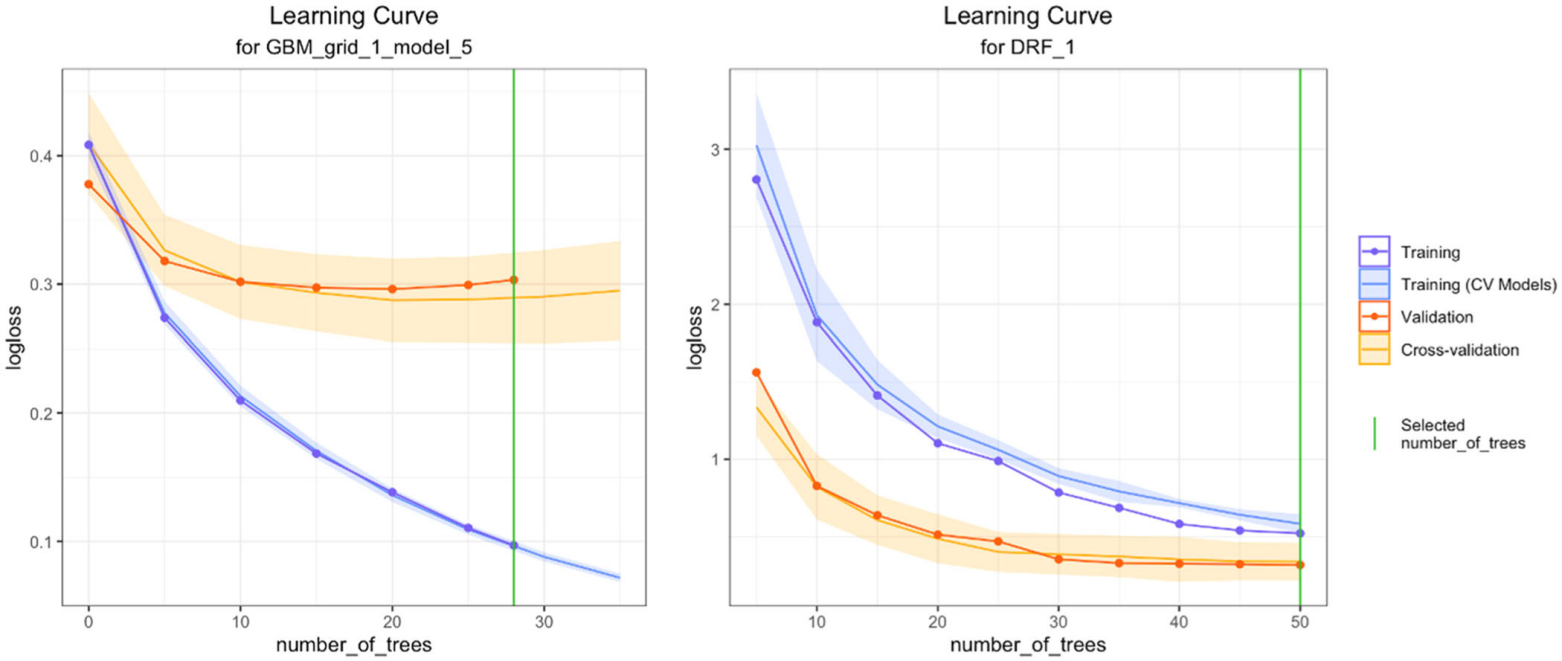
The learning curves of the two ensemble models.

The learning curves for the two models are presented in Figure 1. By examining the overall trends of the training and validation curves for these models, it was observed that the DRF model exhibits better performance in terms of decreasing the difference between the two curves. Conversely, the GBM model demonstrates an initial divergence between the curves during the early stages of the learning process. While the performance metrics for these models are comparable in our testing data set, it is anticipated that in a larger testing data set, the DRF model would outperform the GBM model.

## 4 DISCUSSION

In the medical field, ML is often employed to create predictive models for complex data sets, due to its ability to handle high‐dimensional relationships between features. In this study, we aimed to predict the occurrence of 1‐year MACEs of the STEMI patients who were treated with primary PCI, using four ML models: Gradient boosting, DRF, DL, and LR. The performance of each model was then compared, with DRF model exhibiting the highest AUC value, surpassing that of the other models.

The Global Registry of Acute Coronary Event (GRACE) and the Thrombolysis in Myocardial Infarction (TIMI) risk scores are currently the most widely used scoring systems for predicting the outcomes of STEMI patients.^6^, ^7^, ^8^, ^9^, ^10^, ^11^ However, despite their popularity, these score systems have limitations as they do not include important predictors such as echocardiographic parameters and laboratory data, which may reduce their effectiveness in subgroups of patients. In such cases, ML‐based models that rely on electronic medical records and artificial intelligence can provide a more comprehensive approach to outcome prediction. These ML‐based models can capture a greater number of variables and complex relationships between features, thereby improving the accuracy and specificity of STEMI outcome prediction.

In 2023, Kasim reported that ML models developed using ML feature selection demonstrated superior performance compared to the conventional risk score, TIMI (AUC: 0.81). Among the individual ML models, SVM Linear with selected features exhibited the best performance, outperforming even the best‐performing stacked EL model (AUC: 0.934, confidence interval [CI]: 0.893–0.975 vs AUC: 0.914, CI: 0.871–0.957). Additionally, the women‐specific model demonstrated better performance than the general non‐gender‐specific model (AUC: 0.919, CI: 0.874–0.965). These findings indicate the potential for ML models to improve risk stratification for patients with cardiovascular disease and may contribute to personalized medical decision‐making.^12^, ^13^

In a noteworthy study by Aziz et al., it was demonstrated that ML algorithms have superior predictive performance over the traditional TIMI score system. Notably, the TIMI score system underestimates the risk of mortality. The study showed that 90% of nonsurviving patients were classified as high risk (>50%) by the ML algorithm, in contrast to 10%–30% of nonsurviving patients by TIMI. These results indicate that ML algorithms may provide a more accurate and reliable risk stratification approach in clinical settings.^14^

The study conducted by Bei Shi revealed that in previous studies, ML methods had displayed superior predictive ability for short‐term mortality after STEMI, with XGBoost exhibiting better performance than other ML models in patients with anterior wall STEMI. Gradient boosted tree (GBT) methods, including XGBoost, RF, and CatBoost, demonstrated similar AUC values according to the study conducted by Zhixun Bai.^15^ However, following model optimization, the CatBoost model displayed more accurate predictive ability in the latter study conducted by Bei Shi. The CatBoost algorithm, one of the three mainstream GBT ML methods, is based on an asymmetrical decision tree algorithm (oblivious trees) with only a few parameters and supports class variables while achieving high accuracy. Its primary focus is to address the efficient and reasonable handling of category features. Moreover, a new method was proposed to account for gradient deviation (gradient bias) and prediction partial (prediction shift) problems to improve the algorithm's accuracy and generalization ability. As a new algorithm released in 2017, CatBoost can effectively prevent overfitting and account for category features in clinical practice, which has aroused widespread interest due to its high training accuracy. Recent studies have demonstrated its high accuracy in predicting short‐term mortality after STEMI.

According to the study conducted by Sherazi et al., their soft voting ensemble (SVE) exhibited superior performance when compared to other ML models such as RF and GBMs. The observed difference in performance was statistically significant, indicating the potential of SVE as a powerful tool for predictive modeling.^16^

In 2021, Lee et al. conducted a survey of approximately 14 000 patients with STEMI and non‐STEMI (NSTEMI) to evaluate the performance of ML models in predicting patient mortality compared to traditional models (TM). The study found that ML models exhibited comparable performance to TM in predicting mortality for STEMI patients at in‐hospital, 3‐ and 12‐month follow‐up intervals. In contrast, the ML models outperformed TM in predicting mortality for NSTEMI patients, with AUCs of 0.889, 0.849, and 0.860 for in‐hospital, 3‐ and 12‐month mortality, respectively, compared to AUCs of 0.873, 0.795, and 0.808 for TM. These findings suggest that ML models may be a valuable tool for predicting mortality in NSTEMI patients.^17^

One of the main advantages of RF models is their ability to handle larger data inputs, nonlinear variables, and variable interactions, and avoid overfitting. In our study, we developed a risk stratification model for the mortality of patients with acute myocardial infarction using DL from a large perspective national registry. The results of the accuracy test showed that the deep‐learning model had excellent performance in predicting prognosis and outperformed the conventional risk‐prediction model.

### 4.1 Study limitations

There were some limitations to this study that should be considered when interpreting the results. First, the fact that it was a single‐center study may affect the generalizability of the findings. However, it is important to note that the Tehran Heart Center is a highly regarded referral hospital in Iran, with a diverse patient population. Second, the retrospective nature of this study poses concerns in the interpretation of its results and extending the applicability of the prediction models. Third, the sample size used in this study was not large enough to allow for precise testing and training of the model. The low event rate meant that the power of the study was not high enough, and further research with a larger sample size is required in this field. At last, the lack of external validation is another limitation. The absence of external validation, wherein model performance is assessed on previously unseen data, can lead to reduced generalizability. In ML studies, particularly those involving complex models, the lack of external validation can result in overfitting, where the model fits noise in the training data rather than capturing underlying patterns. Incorporating diverse validation data sets, using cross‐validation techniques, and conducting replication studies are some strategies to address this problem. As our clinic is one of the few national referral centers with a well‐structured registry system for primary PCI patients in the country, we were unable to obtain other structured data for accurate external validation of our models; nevertheless, we evaluated our models with a testing data set that was not seen during the learning process of the models.

## 5 CONCLUSION

Our study has led us to the conclusion that ML‐based models can be an effective tool for identifying STEMI patients who are at the highest risk of developing adverse events during follow‐up. The field of personalized medicine is one that stands to benefit greatly from these algorithms, as they can aid physicians in detecting high‐risk patients earlier and taking appropriate preventive measures. By leveraging the predictive power of ML algorithms, physicians can make more informed decisions about patient care, potentially leading to improved outcomes.

## AUTHOR CONTRIBUTIONS

**Saeed Tofighi**: Design, concept, writing the manuscript, machine learning and statistical analysis. **Hamidreza Poorhosseini, Yaser Jenab, Mohammad Alidoosti, Mohammad Sadeghian, and Mehdi Mehrani**: Scientific supervision and critical revisions. **Zhale Tabrizi and Parisa Hashemi**: Writing and revising the manuscript. All authors contributed to the article and approved the submitted version.

## CONFLICT OF INTEREST STATEMENT

The authors declare that the research was conducted in the absence of any commercial or financial relationships that could be construed as a potential conflict of interest.

## Data Availability

The data that support the findings of this study are available on request from the corresponding author (ST). The data are not publicly available due to containing information that could compromise research participant privacy. Requests to access these data sets should be directed to ST, saeedtofighi69@gmail. com.

## References

[1] Kristensen SD , Laut KG , Fajadet J , et al. Reperfusion therapy for ST elevation acute myocardial infarction 2010/2011: current status in 37 ESC countries. Eur Heart J. 2014;35(29):1957‐1970. 24419804 10.1093/eurheartj/eht529

[2] Goldstein BA , Navar AM , Carter RE . Moving beyond regression techniques in cardiovascular risk prediction: applying machine learning to address analytic challenges. Eur Heart J. 2017;38(23):1805‐1814. 27436868 10.1093/eurheartj/ehw302 PMC5837244

[3] Natekin A , Knoll A . Gradient boosting machines, a tutorial. Front Neurorobot. 2013;7:21. 24409142 10.3389/fnbot.2013.00021 PMC3885826

[4] Biau G , Scornet E . A random forest guided tour. Test. 2016;25:197‐227.

[5] Dreiseitl S , Ohno‐Machado L . Logistic regression and artificial neural network classification models: a methodology review. J Biomed Inf. 2002;35(5‐6):352‐359. 10.1016/s1532-0464(03)00034-0 12968784

[6] Antman EM , Cohen M , Bernink PJLM , et al. The TIMI risk score for unstable angina/non–ST elevation MI: a method for prognostication and therapeutic decision making. JAMA. 2000;284(7):835‐842. 10938172 10.1001/jama.284.7.835

[7] Morrow DA , Antman EM , Charlesworth A , et al. TIMI risk score for ST‐elevation myocardial infarction: a convenient, bedside, clinical score for risk assessment at presentation: an intravenous nPA for treatment of infarcting myocardium early II trial substudy. Circulation. 2000;102(17):2031‐2037. 11044416 10.1161/01.cir.102.17.2031

[8] Morrow DA . Application of the TIMI risk score for ST‐elevation MI in the National Registry of Myocardial Infarction 3. JAMA. 2001;286(11):1356‐1359. 11560541 10.1001/jama.286.11.1356

[9] Tang EW , Wong C‐K , Herbison P . Global Registry of Acute Coronary Events (GRACE) hospital discharge risk score accurately predicts long‐term mortality post acute coronary syndrome. Am Heart J. 2007;153(1):29‐35. 17174633 10.1016/j.ahj.2006.10.004

[10] D'Ascenzo F , Biondi‐Zoccai G , Moretti C , et al. TIMI, GRACE and alternative risk scores in acute coronary syndromes: a meta‐analysis of 40 derivation studies on 216,552 patients and of 42 validation studies on 31,625 patients. Contemp Clin Trials. 2012;33(3):507‐514. 22265976 10.1016/j.cct.2012.01.001

[11] Silveira DS , Jaeger CP , Hatschbach L , Manenti ERF . Validation of TIMI risk score for STEMI. Int J Cardiovasc Sci. 2016;29(3):189‐197.

[12] Kasim S , Rudin PNFA , Malek S , et al. In‐hospital mortality prediction using machine learning and stacked ensemble learning of Asian women with ST‐elevation myocardial infarction (STEMI). *Research Square*. 2023. 10.21203/rs.3.rs-2611510/v1

[13] Kasim S , Malek S , Ibrahim KS , Kumar DS . Applying an interpretive machine learning algorithm to predict in‐hospital mortality in elderly Asian patients with acute coronary syndrome (ACS). Eur Heart J. 2023;44(suppl 1):ehac779125.

[14] Aziz F , Malek S , Ibrahim KS , et al. Short‐and long‐term mortality prediction after an acute ST‐elevation myocardial infarction (STEMI) in Asians: a machine learning approach. PLoS One. 2021;16(8):e0254894. 34339432 10.1371/journal.pone.0254894 PMC8328310

[15] Bai Z , Hu S , Wang Y , et al. Development of a machine learning model to predict the risk of late cardiogenic shock in patients with ST‐segment elevation myocardial infarction. Ann Transl Med. 2021;9(14):1162. 34430603 10.21037/atm-21-2905 PMC8350690

[16] Sherazi SWA , Bae J‐W , Lee JY . A soft voting ensemble classifier for early prediction and diagnosis of occurrences of major adverse cardiovascular events for STEMI and NSTEMI during 2‐year follow‐up in patients with acute coronary syndrome. PLoS One. 2021;16(6):e0249338. 34115750 10.1371/journal.pone.0249338 PMC8195401

[17] Lee W , Lee J , Woo S‐I , et al. Machine learning enhances the performance of short and long‐term mortality prediction model in non‐ST‐segment elevation myocardial infarction. Sci Rep. 2021;11(1):12886. 34145358 10.1038/s41598-021-92362-1 PMC8213755

